# Diffractive properties of imaginary-part photonic crystal slab

**DOI:** 10.1186/1556-276X-7-335

**Published:** 2012-06-21

**Authors:** Haoxiang Jiang, Jingfeng Liu, Gengyan Chen, Xue-Hua Wang

**Affiliations:** 1 State Key Laboratory of Optoelectronic Materials and Technologies, School of Physics and Engineering, Sun Yat-sen University, Guangzhou, 510275, China; 2 College of Science, South China Agriculture University, Guangzhou, 510642, China

**Keywords:** Imaginary-part photonic crystal, Diffractive efficiency, Red shift, Active material

## Abstract

The diffraction spectra of imaginary-part photonic crystal (IPPC) slabs are analyzed using the scattering-matrix method. By investigating the thickness dependence of the diffraction, we find a remarkable red shift of central wavelength of the diffraction spectrum, which obviously distinguishes from the phenomenon of spectral hole. We observe that diffraction efficiency can be enhanced more than 20-fold by optimizing the geometry parameters. These imply that the diffraction spectra of the IPPC slab can be controlled at will and used to guide the design to achieve useful nanoscale devices.

## Background

Photonic crystals (PCs) [1,2] are composite nanostructures in which a periodic modulation of refractive index forms photonic bandgaps of frequencies where light propagation is fully suppressed. PCs can manipulate not only the emission of light [3-5], but also the propagation of light, the prominent examples of which are PC slab waveguides [6-9] and resonant gratings [10-12].

In most cases, PCs are composed of mediums with different real dielectric constants. However, by embedding an absorbing medium into the structure, novel physical phenomena and new types of optoelectronic devices can be created [13,14]. Recently, Li et al. [15] propose an innovative type of PCs, named imaginary-part photonic crystals (IPPCs). The new type of PCs is composed of a selected dielectric medium with and without doping agent of resonant absorption medium. This new structure provides a frequency-dependent character: the IPPCs have periodic modulation of dielectric constant near the resonant frequency, but off the resonant frequency, they become ordinary structures with uniform dielectric constant. In a word, the fundamental properties of IPPCs result from the doping agent.

Since active mediums always have resonant enhancement of nonlinear effect, fast response speed, and low working threshold, many intense applications of IPPCs, such as fabrication of *resonantly absorbing waveguide arrays*[16,17] and *inverted nonlinear photonic crystals*[18], have been reported. Lately, the IPPCs were found to have potential applications in display industry because of their high efficiency of color separations.

It is well known that diffraction control of light field is very important in holographic lithography, and the IPPCs have been reported to be sensitive in controlling the diffraction efficiency. Studying the diffraction properties and finding optimized diffraction efficiency in the IPPCs become an interesting issue. Some diffraction properties have been reported by Li et al. [15] and Feng et al. [17]; they present the wavelength-dependent diffraction efficiency in simple lattice structures and adopt the paraxial approximation method. Up to now, the detail dependences of diffraction efficiency on geometry parameters of the IPPCs have not been reported yet. Moreover, developing a rigorous method to exactly and efficiently investigate the fascinating characteristics of the IPPCs is necessary.

In this paper, we develop and apply the scattering-matrix method (SMM) [19] to exactly analyze the diffractive properties of two-dimensional (2D) IPPC slabs. With increasing thickness, we find an interesting phenomenon that the central wavelength of the diffraction spectrum shifts towards to the red end of the spectrum, which distinguishes from the phenomenon of spectral hole [17]. The roles and competition of imaginary and real part contributions are investigated to understand these phenomena. Besides, the dependence of diffraction efficiency on the geometry parameters is investigated to find remarkable enhancement effect of diffraction around resonant wavelength. It offers us an approach to finely control the diffraction spectra of the IPPC slabs at will. More than 20-fold enhancement in the maximum diffraction efficiency can be reached.

## Methods

### Theory and formulation

**Figure 1:**
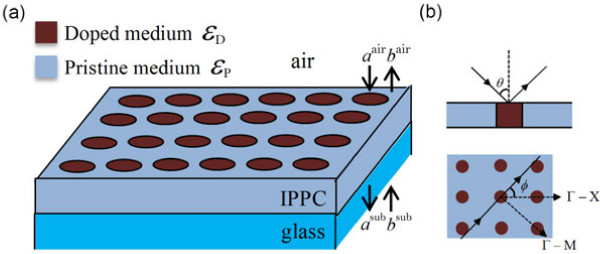
**The structure of 2D IPPC slab.** (**a**) Circular rods in square lattice on glass substrate. (**b**) Schematic and plan view of the structure, showing the polar angle *θ* and the azimuth angle *ϕ* of incident light and the high symmetry along *Γ*−X and *Γ*−M directions.

We consider the IPPC slabs on a glass substrate with infinite thickness and incident light with polar angle *θ* and azimuth angle *ϕ*, as shown in Figure 1. We define the dielectric constant of the doped medium as *ε*_D_ = *ε*_Dr_ + *iε*_Di_ and the pristine medium as *ε*_P_ = *ε*_Pr_ + *iε*_Pi_, where *ε*_Dr_ ( *ε*_Pr_) and *ε*_Di_ ( *ε*_Pi_) are the real part and the imaginary part of *ε*_D_ ( *ε*_P_), respectively.

According to the diffraction theory, the *n*th-order diffraction efficiency *η*_*n*_ is defined as the ratio of the *n*th-order Fourier transform component of average diffraction outgoing energy flux $P_{⊥}^{\text{sub}}$ to the incident average energy flux $P_{⊥}^{\text{air}}$. It can be calculated by

(1) $$\eta_{n}={\left(P_{⊥}^{\text{sub}}\right)}_{n}/P_{⊥}^{\text{air}}).$$

Average energy flux can be calculated by $\bar{P}=\frac{1}{2}\text{Re}\left(E^{*}\times H\right)$. We can easily obtain $P_{⊥}^{\text{air}}$ from the electromagnetic fields of incident light **H**_**0**_ and **E**_**0**_, and $P_{⊥}^{\text{sub}}$ according to the rigorous SMM. Moreover, the SMM can be adopted to calculate diffraction because it analyzes problems in Fourier space. The outgoing diffractive component of the *n*th order can be obtained by

(2) $${\left(P_{⊥}^{\text{sub}}\right)}_{n}=-e_{y,G_{n}}^{\text{sub}}h_{x,G_{n}}^{\text{sub}}+e_{x,G_{n}}^{\text{sub}}h_{y,G_{n}}^{\text{sub}},$$

where $e_{x}^{\text{sub}}$, $e_{y}^{\text{sub}}$, $h_{x}^{\text{sub}}$, and $h_{y}^{\text{sub}}$ are the in-plane electric and magnetic Fourier components which are obtained by the Fourier expansion of *E*_*x*_, *E*_*y*_, *H*_*x*_, and *H*_*y*_, respectively, in the glass substrate layer; **G**_**n**_ is a reciprocal lattice vector which corresponds to the *n*th diffraction order. Since $e_{x}^{\text{sub}}$ and $e_{y}^{\text{sub}}$ can be obtained by $h_{x}^{\text{sub}}$ and $h_{y}^{\text{sub}}$, we focus on magnetic Fourier components.

On the other hand, for every single layer in *z* plane, magnetic Fourier space vectors *h*_*x*_(*z*) and *h*_*y*_(*z*) can be expanded in terms of the propagating modes which are eigenvectors of the propagation eigen problem

(3) $$h_{∥}\left(z\right)\equiv \left(\begin{array}{c} h_{x}\left(z\right)\\ h_{y}\left(z\right) \end{array}\;\right)=\Phi \left[\hat{\text{f}}\left(z\right)a+\hat{\text{f}}\left(d-z\right)b\right],$$

where the *n*th eigenvector is the *n*th column vector of matrix *Φ*; $\hat{f}\left(z\right)$ is a diagonal matrix with $\hat{f}{\left(z\right)}_{\mathrm{nn}}=e^{iq_{n}z}$, here, *q*_*n*_ is the *n*th eigenvalue of the propagation eigen problem in this layer; *d* is the thickness of this layer; *a* and *b* are a couple of vectors whose coefficients correspond to the amplitudes of forward and backward going wave as shown in Figure 1, respectively. The details of the method can be found in [19].

For the incident layer, the vector $h_{∥}^{\text{air}}$ should be separated to incident part $h_{∥,\text{inc}}^{\text{air}}=\Phi^{\text{air}}a^{\text{air}}$ and reflective part $h_{∥,\text{ref}}^{\text{air}}=\Phi^{\text{air}}b^{\text{air}}$(for the incident plane, $\hat{f}\left(z\right)=\hat{f}\left(0\right)=1$). The amplitude vector of the incident light *a*^air^ can be obtained by the Fourier expansion of incident **H**_**0**_ as

(4) $$a^{\text{air}}={\left(\Phi^{\text{air}}\right)}^{-1}h_{∥}^{\text{air}}.$$

Because the electromagnetic field at the interfaces between two layers satisfies boundary conditions, the vectors of the transmission amplitude can be calculated by the S-matrix which relates *a*^sub^ and *b*^air^ to *a*^air^ and *b*^sub^ as [19]

(5) $$\left(\begin{array}{c} a^{\text{sub}}\\ b^{\text{air}} \end{array}\;\right)=S\left(\begin{array}{c} a^{\text{air}}\\ b^{\text{sub}} \end{array}\;\right)\equiv \left(\begin{array}{c} S_{11}\;\;S_{12}\\ S_{21}\;\;S_{22} \end{array}\;\right)\left(\begin{array}{c} a^{\text{air}}\\ b^{\text{sub}} \end{array}\;\right).$$

Since there is no incident light from the glass substrate *b*^sub^ = 0, the transmission amplitude becomes *a*^sub^ = *S*_11_*a*^air^. Combining with Equations 3 and 4, we can obtain the magnetic Fourier expansion (for the outgoing plane, $\hat{f}\left(z\right)=\hat{f}\left(0\right)=1$)

(6) $$h_{∥}^{\text{sub}}=\Phi^{\text{sub}}S_{11}{\left(\Phi^{\text{air}}\right)}^{-1}h_{∥}^{\text{air}}.$$

Now, we can investigate any order of diffraction efficiency, when we select the corresponding component of $h_{∥}^{\text{sub}}$ to calculate ${\left(P_{⊥}^{\text{sub}}\right)}_{n}$. Furthermore, this method also can be extended to anisotropic medium [20].

The in-plane wave vector of transmission diffraction, $k_{∥}^{\text{sub}}$, can be obtained by

(7) $$k_{∥}^{\text{sub}}=k_{0}\sin\theta +n_{1}t_{1}+n_{2}t_{2},$$

where **k**_**0**_ is the wave vector of incident light, **t**_**1**_ and **t**_**2**_ are the reciprocal primitive vectors of a periodic structure, *n*_1_ and *n*_2_ are integers determining the diffraction propagation direction. This implied that **G**_**n**_ = *n*_1_**t**_**1**_ + *n*_2_**t**_**2**_. It is noticeable that there are four first-order diffractions in 2D PC slabs corresponding to ( *n*_1_, *n*_2_) = (0,±1) and (±1,0). In the case of normal incident light (*θ* = 0°), because of the symmetry, four diffraction efficiencies of the first order are equivalent, so that one of them can stand for first-order diffraction efficiency (FODE).

## Results and discussions

In this section, we investigate not only the red shit of the central wavelength, but also the influence of different geometry parameters with normal incident light and different incident angle. In the following calculation, 625 plane waves are used to guarantee the favorable convergence and high accuracy.

**Figure 2:**
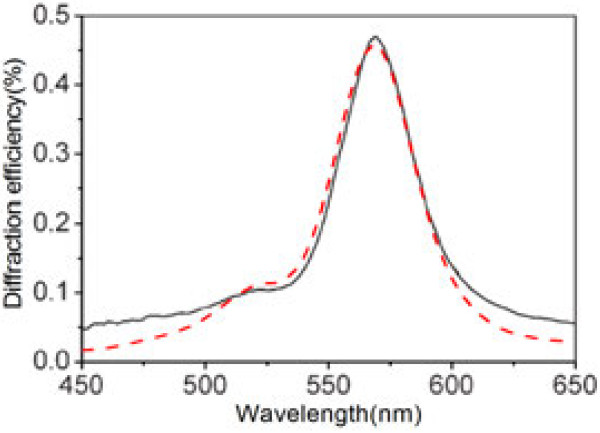
**Comparing experimental and numerical results.** Experimental (black solid line) and numerical (red dash line) diffraction spectra of the IPPC slab.

To validate the theoretical analysis methods, we firstly consider the identical structure of IPPC as [15], with the dielectric constant of the pristine medium *ε*_Pr_ = 2.62, *ε*_Pi_ = 0 and resonant absorption wavelength *λ*_0_ = 564 nm of doping agent. Good agreement of first-order diffraction efficiency is shown, between our simulation and the experimental result offered by [15], as illustrated in Figure 2. So, SMM is verified as a suitable method to further analyze and investigate characters of IPPCs.

### The red shift of resonant diffraction

**Figure 3:**
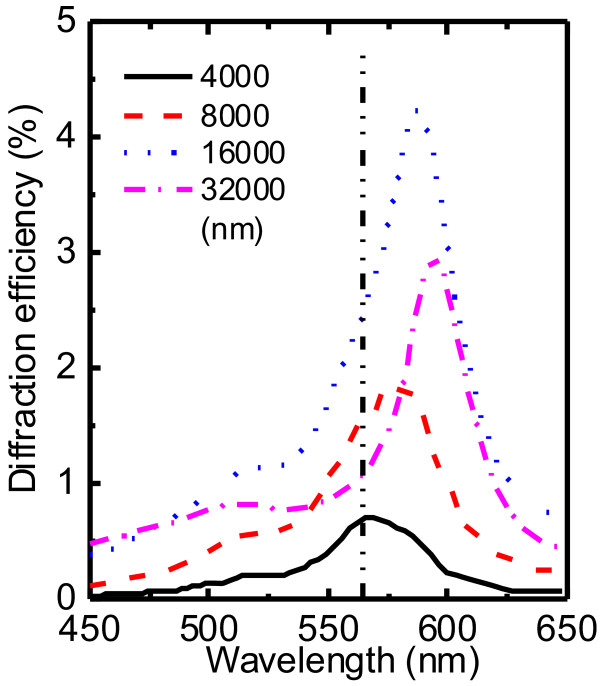
**Red shift of central wavelength.** The FODE spectra of the IPPC slab with circular rod in square lattice, with different slab thickness. The dash-dot-dot vertical line is the resonant absorption wavelength *λ*_0_ of doping agent.

We now apply SMM to investigate the influence of the slab thickness on the diffraction by keeping the other parameters unchanged. The results are shown in Figure 3. It is very interesting to find red shift phenomenon of central wavelength of the diffraction spectrum from resonant absorption wavelength *λ*_0_ of doping agent when varying the slab thickness. To explain this interesting phenomenon, the individual contributions from real and imaginary parts of the dielectric constant to the diffraction is investigated by neglecting the Kramers-Kronig relation [21].

**Figure 4:**
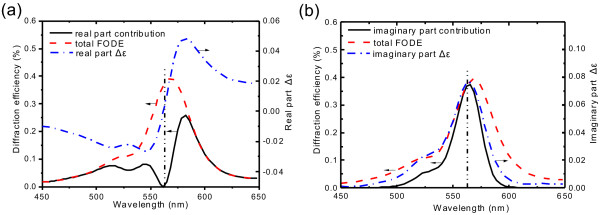
**Real and imaginary part contributions to total diffraction efficiency.** Contributions to diffraction efficiency by (**a**) real part *Δ*_*ε*_ and (**b**) imaginary part *Δ*_*ε*_ of the IPPC slab, independently. The blue dash-dot lines denote the real/imaginary part *Δ*_*ε*_. The black solid lines denote real/imaginary part contributions. The red dash lines denote total FODE calculated by using original *ε*_D_ and *ε*_P_. The dash-dot-dot vertical line is the resonant absorption wavelength *λ*_0_ of doping agent.

Firstly, we consider identical IPPC as [15] whose thickness is 2.6 *μ*m and filling factor is 15.2*%*. We define *Δ*_*ε*_ = *ε*_D_ − *ε*_P_. For investigating the real part contribution, we assume *ε*_Di_ = *ε*_Pi_, where only the real part of *Δ*_*ε*_ exists. The spectrum of real contribution is shown in Figure 4a. The FODE curve of the real part contribution synchronously responds to the absolute value of the real part *Δ*_*ε*_. Furthermore, around *λ*_0_, the real part contribution vanishes because the real part *Δ*_*ε*_ gets through zero from negative minimum to positive maximum sharply. Secondly, we turn to investigate the influence of the imaginary part on diffraction efficiency by assuming *ε*_Dr_ = *ε*_Pr_, then the *Δ*_*ε*_ only changes in the imaginary part. The FODE curve of the imaginary part contribution is in the same pace with the imaginary part *Δ*_*ε*_, as shown in Figure 4b. At *λ*_0_, the imaginary part contribution reaches its maximum since the imaginary part *Δ*_*ε*_ reaches its maximum.

It is noticeable that the imaginary part contribution takes charge of total FODE curve at *λ*_0_ while the real part contribution dominates around *λ*_0_ and results in the red shift of central wavelength of total FODE. Because the imaginary part of the dielectric constant not only contributes diffraction but also absorbs the propagating light, the ratio of real part contribution to imaginary part contribution grows with increasing thickness and the red shift phenomenon becomes remarkable (Figure 3).

Another interesting phenomenon is that an diffraction spectral hole appears at the absorption center at thickness larger than about 10*μ*m [17], which results from the weakness of imaginary part contributions in the competition in the cases with large thickness. It is noticeable that the red shift phenomenon appears in the structures with small filling factor, while the spectral hole phenomenon appears in the ones with large filling factor.

In a word, the red shift phenomenon and the spectral hole phenomenon can be reasonably explained by the competition between real and imaginary part contributions, and the specified central wavelength of diffraction spectral can be obtained by modulating the thickness of IPPC.

### Remarkable enhancement effect of diffraction

To achieve lager diffraction efficiency, we investigate the dependence of diffraction on each geometry parameter including lattice constant, slab thickness, filling factor, lattice type, and rod shape at the resonant absorption wavelength *λ*_0_ of doping agent with normal incident light. We focus on the IPPC slabs with different rod shapes including circular rod (CR), square rod (SR), and hexagon rod (HR), and with different lattice types including square lattice (SL), triangle lattice (TL), and honeycomb lattice (HL) [22]. Furthermore, the dependence of diffraction on incident angler and polarization is discussed in detail. Finally, we obtain significant enhancement in diffraction efficiency.

**Figure 5:**
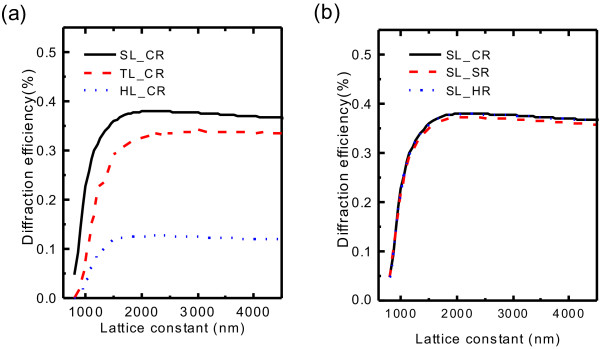
**Dependence of diffraction on lattice constant.** By keeping a filling factor of 15.2*%*, the FODE at *λ*_0_ with (**a**) circular rod in different lattice types and (**b**) different rod shapes in square lattice.

First of all, we investigate the dependence of diffraction on the lattice constant at *λ*_0_, as shown in Figure 5a, b. With the increasing lattice constant, FODE rises rapidly until reaching its maximum around 2,000 nm, where lattice constant is several times larger than incident wavelength, and then FODE declines slightly. Therefore, a sufficiently large lattice constant is desirable to obtain high FODE. In practice, a large lattice constant may bring convenience to fabrication but lead to difficulties for detection and application, since the diffraction angle will decrease while enlarging the lattice constant.

Figure 5a shows that lattice type plays much more important role on the diffraction. Square lattice and triangle lattice result in much higher FODE than honeycomb lattice does, so they are widely adopted experimentally. In contrast, the shape of rods bears little or no relationship to the FODE. The FODEs at resonant wavelength of the three rod shapes for the square lattice type are almost identical, as shown in Figure 5b, which is also evident for the other two lattice types. So, the circular rod is widely adopted due to easy fabrication.

**Figure 6:**
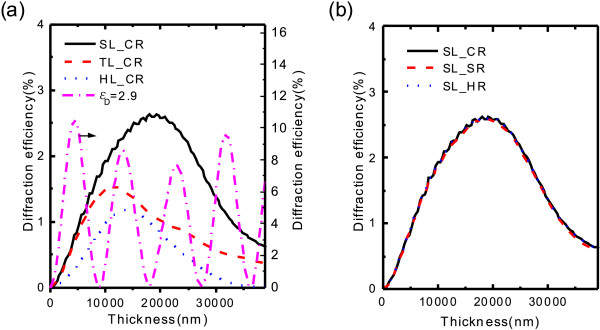
**Dependence of diffraction on thickness.** By keeping a filling factor of 15.2*%*, the FODE at *λ*_0_ with (**a**) circular rod in different lattice types and (**b**) different rod shapes in square lattice. The magenta dash-dot line in (a) corresponds to FODE which is calculated by assuming *ε*_D_ = 2.9 with square lattice.

Secondly, the thickness of the IPPC slab can strongly influence the diffraction efficiency, as shown in Figure 6a, b. For each lattice type and rod shape, the FODE can reach its maximum at certain slab thickness, like the one-dimensional resonantly absorbing waveguide array in [17]. When the IPPC slab is very thin, the propagation light almost transmits through in zero-order diffraction. In contrast, when the IPPC slab is sufficiently thick, due to the absorption of the medium, the propagation light cannot pass through the slab. So, the maximum of FODE appears at an appropriate thickness in IPPC slabs. This characteristic is quite different from that of the PC slab composed with the non-absorption medium, illustrated by the magenta dash-dot line in Figure 6a, whose diffraction spectrum oscillates along with thickness. For the non-absorptive-medium PC slab, large contrast of dielectric constant between two mediums would be selected to obtain a remarkable diffraction effect. However, for the IPPC slab, the diffraction efficiency is hard to enhance because of the absorption of light and the small difference of dielectric constant between pristine medium and doped medium with small saturated concentration of doping. Moreover, Figure 6a, b shows the FODEs of different lattice types with circular rod and different rod shapes in square lattice, which can be concluded again that the lattice type plays an important role and the rod shape takes unimportant part in the diffraction. Square lattice results the highest FODE in all these lattice types.

**Figure 7:**
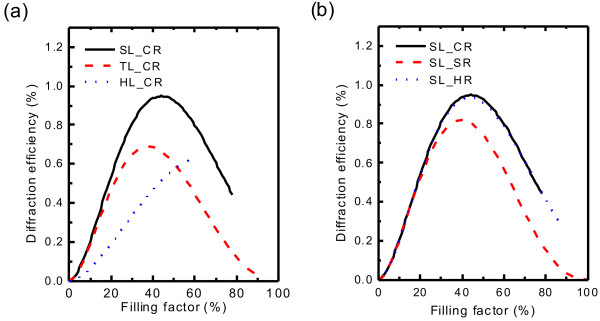
**Dependence of diffraction on filling factor.** The FODE at *λ*_0_ with (**a**) circular rod in different lattice types and (**b**) different rod shapes in square lattice.

Thirdly, filling factor is a sensitive parameter to influence the FODE of the IPPC slab, illustrated in Figure 7a, b. It can be found that FODE increases first, but after reaching the maximum, it decreases fast. Besides, the lattice types affect FODE strongly, and the IPPC slab in square lattice results in the highest diffraction efficiency; rod shapes still have little influence on FODE, except for the IPPC slab with square rod in square lattice due to its lowest symmetry.

From the above investigation, we obtain the dependent properties of diffraction on geometry parameters and conclude that lattice type strongly influences the FODE while the influence of rod type is negligible. Moreover, among the three lattice types, square lattice is found to be the best structure for large diffraction efficiency.

Fourthly, we turn to reveal the relation between incident angle and diffraction efficiency of the IPPC slab with circular rod in square lattice by varying *θ* and *ϕ* of transverse magnetic (TM) and transverse electric (TE) polarization incident light, respectively.

**Figure 8:**
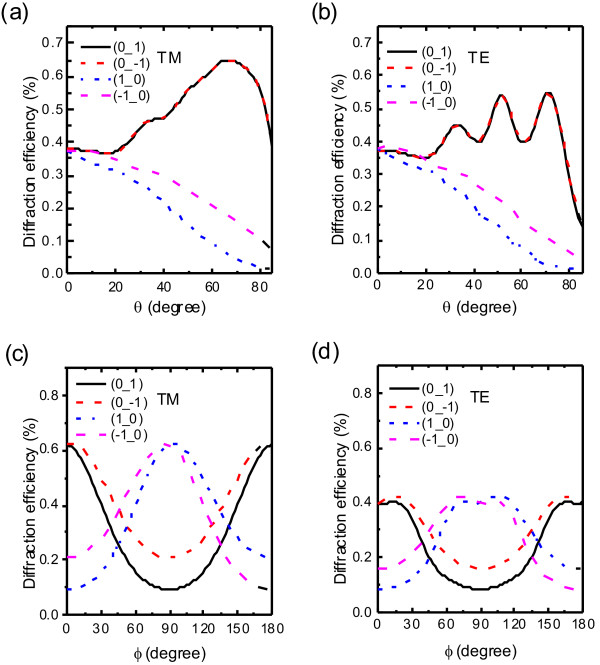
**Dependence of four first-order diffractions on incident angle and polarization.** Four first-order diffractions (0,1), (0,−1), (1,0), and (−1,0) with TM and TE incident light on incident angle. (**a**) and (**b**) show the FODE at *λ*_0_ with incident light in the *Γ*−X direction with different *θ*, while (**c**) and (**d**) show it with different *ϕ* at *θ*= 60°.

When *θ* ≠ 0°, the four diffraction efficiencies of the first order are no longer the same. Figure 8a,b shows the FODE of the resonant wavelength as a function of *θ*, from which we can find that the energy of diffraction redistributes. With increasing *θ*, the curves fall into two categories: those of (±1,0) decrease tonelessly, while those of (0,±1) tend to increase and then decrease dramatically with TM/TE polarization incident light after large *θ*. On the other hand, FODEs of the four first-order diffractions as a function of *ϕ* reach their maximum and minimum alternately, illustrated in Figure 8c, d. The incident light along the *Γ*−X direction leads to a large difference among the first-order diffractions, while that along the *Γ*−M direction leads to much smaller in square lattice. So, choosing a proper polar angle is the key to obtain larger FODE, and adjusting the azimuth angle is necessary to select which first-order diffraction efficiency to be enhanced.

**Figure 9:**
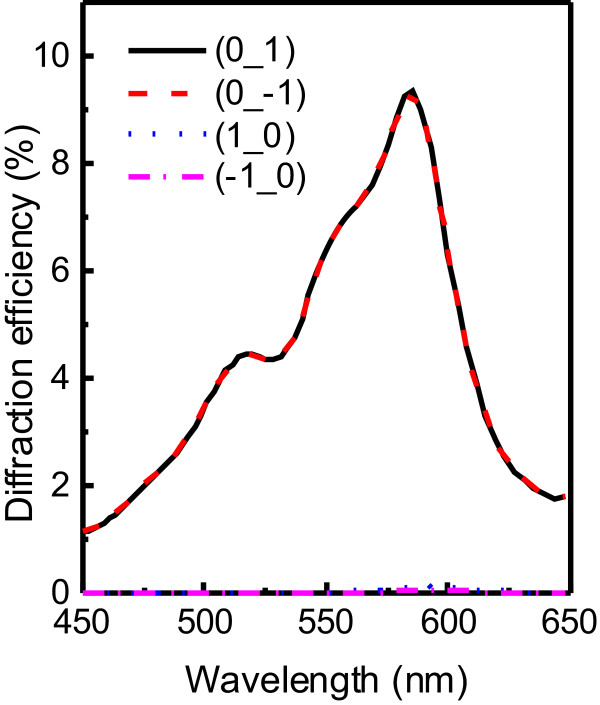
**Optimized diffraction spectrum.** Spectrum of FODE with a lattice constant of 4*μ*m, a thickness of 20*μ*m, a filling factor of 15.2*%*, *θ* = 60°, *ϕ* = 0°, and circular rod in square lattice with TM polarization incident light.

Finally, by modulating these geometry parameters and incident angle, we can obtain more than 20-fold FODE enhancement than that reported in [15] in a 2D IPPC slab with TM polarization incident light, as shown in Figure 9. It is noticeable that the large thickness and filling factor of the slab not only strongly enhance the diffraction efficiency, but also lead to deformation with red shift or spectral hole phenomenon. We select a filling factor of 15.2*%* to avoid spectral hole phenomenon.

It is clear that we can obtain remarkable enhancement of diffraction efficiency and desirable shape of diffraction spectrum by utilizing the dependences of diffraction on geometry parameters and incident angle.

## Conclusions

We employ and develop the scattering-matrix method to investigate the diffractive characteristics of 2D IPPC slabs rigorously. An interesting red shift of central wavelength of the diffraction spectrum in large thickness is observed and explained by analyzing the competition of imaginary and real part contributions. On the other hand, we obtain more than 20-fold enhancement of maximum FODE by investigating the dependences of diffraction on geometry parameters. To obtain remarkably large FODE, a sufficiently large lattice constant and an appropriate thickness and filling factor are necessary. Besides, lattice types play a significant role while rod shapes only slightly influence FODE. Among all lattice types, the highest diffraction efficiency can be obtained by the IPPC slab of square lattice. In the dependence of diffraction on incident angle, polar and azimuth angles can strongly influence and adjust the distribution of four diffraction efficiencies, respectively. As a result, the diffractive properties of IPPC can be used to guide the design to achieve useful nanoscale devices.

## Competing interests

The authors declare that they have no competing interests.

## Authors’ contributions

HXJ did all of the calculations and drafted the manuscript. XHW contributed the idea to this work and drafted the manuscript. JFL and GYC hleped HXJ to do calculations and codrafted the manuscript. All authors read and approved the final manuscript.

## Authors’ information

XHW is a professor in Optics in the School of Physics and Engineering, Sun Yat-sen University, China. He got his Ph.D. degree in 1995 at Shanghai Jiaotong University, China. Then, he spent 2 years as postdoctor in the Institute of Physics, Chinese Academy of Sciences, Beijing. His current research interests mainly focus on nano-optics, quantum optics, quantum computation, and nonlinear optics, especially on the control of strong interaction between light and materials in inhomogeneous electromagnetic nanostructures, such as photonic crystals and nanometal structures. HXJ and GYC are Ph.D. students in the School of Physics and Engineering, Sun Yat-sen University, China. JFL is an assistant professor in the College of Science, South China Agriculture University. He got his Ph.D. degree in 2011 at Sun Yat-sen University, China.
